# Development and Characterization of Edible Films Based on Cassava Starch Modified by Corona Treatment

**DOI:** 10.3390/foods13030468

**Published:** 2024-02-02

**Authors:** Carlos Mauricio Otálora González, Manuel Felix, Carlos Bengoechea, Silvia Flores, Lía Noemí Gerschenson

**Affiliations:** 1 Departamento de Industrias, Facultad de Ciencias Exactas y Naturales, Universidad de Buenos Aires, Intendente Guiraldes 2620, Buenos Aires 1428, Argentina; camaota@yahoo.es (C.M.O.G.); skflores14@gmail.com (S.F.); lia@di.fcen.uba.ar (L.N.G.); 2 Instituto de Tecnología de Alimentos y Procesos Químicos (ITAPROQ), CONICET—Universidad de Buenos Aires, Buenos Aires 1428, Argentina; 3 Departamento de Ingeniería Química, Escuela Politécnica Superior, Universidad de Sevilla, 41011 Sevilla, Spain; mfelix@us.es

**Keywords:** starch, corona treatment, modification, edible films

## Abstract

Corona treatment (CT), a surface treatment widely used in the plastic industry, can be used to alter the properties of cassava starch. In the present work, CT was performed on dry granular starch (DS), water-suspended humid granular starch (HS), and gelatinized starch (GS). Different properties and structural characteristics of treated starches were studied. A lowering in pH was generally observed after CT and the rheological properties depended on the starch presentation. A reinforcement of DS and HS samples after CT was deduced from higher viscosity values in flow assays and viscoelastic moduli, but weak gels were obtained when CT was applied to GS. Changes in the A-type polymorphic structure, as well as a drop in relative crystallinity, were produced by CT for DS and HS. Additionally, changes in O-H and C-O-C FTIR bands were observed. Therefore, CT can be applied for starch modification, producing predominantly cross-linking in the DS and de-polymerization in the HS. Casting films made from the modified DS showed higher tensile strength and lower hydrophilicity, solubility, water absorption capacity, and water vapor permeability. Thus, the DS cross-linking induced by CT improved mechanical characteristics and hydrophobicity in edible films, which can be better used as packaging materials.

## 1. Introduction

Starch, constituted by linear polysaccharide molecules (20 to 25% (*w*/*w*) amylose) and branched molecules (75 to 80% (*w*/*w*) amylopectin), is an important renewable resource for the industry. Amylose and amylopectin naturally and hierarchically assemble into granular forms ranging in size from 1 to 100 μm [1]. Some physicochemical and functional properties of native starches, like their limited solubility, resistance to gel formation, or retrogradation, restrict their direct application for certain applications [2]. Thus, starch can be modified chemically, physically, or enzymatically to broaden its applicability [3]. Chemical methods generally provide high efficiency in starch modification, but they have disadvantages related to cost, chemical waste management, and time consumption [4]. The growing interest in environmental protection requires more eco-friendly processes, such as physical starch modification, which is a fast and non-polluting production technique [5]. Plasma treatment is distinguished as an innovative method for starch modification. Corona electrode discharge treatment is an emerging, non-thermal, high-efficiency, lower-energy, and zero-waste technology that can be used to change the functionality of starch [5,6]. This treatment is often used as a pre-treatment for several industrial applications, such as polymer matrices, plastics, or textiles, resulting in modifications in the surface energy and polarity of the treated material [7].

Corona treatment (CT) is performed on the polymer surface through the action of electrodes that generate an electrical discharge (10–50 kV; 1–4 kHz). This discharge causes a partial ionization of the surrounding atmosphere and produces different energetic species (i.e., electrons, ions, photons, free radicals), collectively known as plasma [7,8]. Plasma reactive species interact with starch molecules, forming new covalent bonds and functional groups, like ether, carbonyl, or carboxyl, as well as forming new compounds [9]. Plasma treatments used for starch modification are generally operated under low-pressure conditions through the application of different plasma source gases (e.g., argon, oxygen, hydrogen, methane, ethylene, ammonia) [6,10,11]. Recently, atmospheric pressure cold plasma (ACP) has been employed for decontamination purposes in the food industry [12,13]. Thus, ACP offers many innovative applications, such as increasing shelf-life, improving seed germination rate, promoting microbial inactivation, altering enzyme activity, adjusting hydrophilic or hydrophobic properties, etching or deposition of thin films, etc. [14]. Depending on the plasma type and conditions used, these treatments can modify starch to obtain different functional properties through specific surface reactions. The modifications can be, for example, surface energy increase, hydrophilicity increase or decrease, oxidation, bond breaking, molecular de-polymerization, cross-linking, and the introduction of new functional groups [6,7,10,15].

Lately, the interest in biodegradable materials has increased due to the non-renewable and mostly non-biodegradable character of petroleum-derived materials. Even if edible films generally present many advantages (e.g., degradability, affordability, recyclability readiness) [12], their technological application has found restrictions because of their inherent poor mechanical and barrier properties, high water sensitivity, low surface functionality, or poor printability and adhesiveness [16]. Hence, different treatments are necessary to improve these features [7,17].

At present, no systematic research report concerning the modification of cassava starch using a corona electrode at atmospheric pressure could be found. Therefore, the goals of the present study were to modify cassava starch using CT, determine the physicochemical, thermal, rheological, and structural characteristics, and study the chemical structures of the modified starch. And, finally, simple edible films based on plasma-modified starch were developed and characterized. Three different forms of starch were considered: two for intact granules (dry and in aqueous suspension) and one as a disrupted granule (gelatinized). Thus, the effects of the polysaccharide presentation on the reactions occurring during CT were investigated.

## 2. Materials and Methods

### 2.1. Materials

Native cassava starch (13.1% moisture) was food-grade (Bernesa S.A., Lomas de Zamora Buenos Aires, Argentina). The starch used was characterized by a degree of purity of 92–98% *w*/*w*. The amylose content was 19 % *w*/*w*, the amylopectin content was 81 % *w*/*w,* and the average molecular weight was 68 × 10^6^ Da for amylopectin and 0.8 × 10^6^ Da for amylose [18]. Analytical-grade glycerol (Biopack, Zárate, Buenos Aires, Argentina) and potassium sorbate (Sigma, St. Louis, MI, USA) were used. Deionized water was employed.

### 2.2. Sample Preparation for Plasma Treatment

Three forms of starch presentation were used: dry (DS), humid (HS) (water-suspended), and gelatinized (GS) starch. For DS, the granule without further treatment was used. The HS was obtained by mixing cassava starch in deionized water at a 1:4 ratio (*w*/*w*) for 4 h at 25 °C using a magnetic stirrer (300 rpm). Regarding GS, a suspension of 5% (*w*/*w*) was made by dispersing 5 g of cassava starch in 95 g of distilled water under mechanical stirring (300 rpm) and the system was heated at a rate of 2 °C/min up to 82 °C. The GS was cooled to 25 °C for 10 min before applying the plasma treatment.

#### Corona Treatment

A lab-scale corona unit (Model BD-20, Electro-Technic Products INC, Chicago, IL, USA) was employed. This unit has a voltage output that could be adjusted from 10,000 to 48,000 volts at frequencies from 4 to 5 MHz. Previous tests were performed to establish the proper treatment time. The DS samples (1 g) were supported on a round glass (5 cm diameter) base fixed to an aluminium square mobile platform (12 cm × 12 cm) and were treated with the 3-inch wire field-effect electrode for 2 min at 5 mm from the sample surface. The samples resting on the movable base were displaced at 1.0 cm/s in different directions (i.e., from right to left and from back to front) to ensure that the whole surface received the treatment conveniently. On the other hand, both the HS and GS were treated with a 2-inch-long electrode, located 5 mm from the sample surface, for 8 min. During the treatment, samples were continuously stirred using a magnetic stirrer (300 rpm). After treatment, the HS and GS samples were dried, the former by centrifugation followed by storage in an oven at 40 °C for 24 h, and the latter through freeze-drying. Both dried samples were eventually milled. Untreated (DS-U, HS-U, and GS-U) and treated (DS-T, HS-T, and GS-T) samples were kept at 4 °C.

### 2.3. Fourier Transform Infrared Spectroscopy (FTIR)

A Jasco FTIR 4200 spectrometer (Jasco Analytical Instrument, Tokyo, Japan) was used to obtain FTIR spectra in a wavenumber range of 400–4000 cm^−1^ at a resolution of 4 cm^−1^ in the transmission mode. The tests were carried out on samples after being pressed into KBr pellets.

### 2.4. Determination of pH, Water Retention Capacity (WRC), and Solubility in Water (S)

The pH was measured for 0.5% *w*/*w* aqueous starch suspension. WRC and S were determined according to Alzate et al. [18]. Briefly, 0.1000 g samples were re-suspended in distilled water (9 g) into pre-weighed centrifuge tubes which were placed in a hot water bath (85 °C, 30 min) and then centrifuged (Centrifuge 5804 R, Eppendorf, Hamburgo, Germany) at 5000 rpm for 15 min at 5 °C. A volume of supernatant (5 mL) was dispensed into weighed Petri dishes and dried in a vacuum oven (100 °C, 24 h). The total solubilized mass of starch was expressed as g of solubilized starch per 100 g of original sample (%). The WRC (water per g of sample) was calculated as the ratio of the weight gain of the swelled pellet and the initial weight of the sample discounted by the corresponding soluble fraction.

### 2.5. Pasting Properties

The pasting behavior of untreated and treated samples was measured with a Modular Compact Rheometer (Model MCR 102e, Anton Paar, Seiersberg, Graz-Umgebung, Austria), which was controlled by the RheoCompass software (21 CFR Part 11, 1.21.805 version, Anton Paar). The starch suspension (5%, *w*/*w*) was prepared by suspending 2.5 g cassava starch in 50 g distilled water and stirring for 5 min before subjecting it to the pasting assay. The racket initially rotated at 960 rpm for 10 s, and then was kept at 160 rpm up to the end of the pasting test. All samples were first kept at 50 °C for 1 min, and then heated up to 95 °C at 6 °C/min and held isothermally for 5 min. Eventually, all samples were cooled to 50 °C at 6 °C/min and held isothermally for 2 min. Parameters recorded were peak viscosity (PV, Pa·s), breakdown viscosity (BV, Pa·s), final viscosity (FV, Pa·s), setback viscosity (SV, Pa·s), and pasting temperature (PT, °C). All measurements were performed in duplicate.

### 2.6. Differential Scanning Calorimetry

A TA Instruments Q20 DSC (TA Instrument, New Castle, DE, USA) was employed for the thermal analysis of the starch samples. Different starch samples (3 mg) were mixed with 9 µL of distilled water, sealed in an aluminium capsule, and equilibrated at room temperature for 24 h before analysis. Thermograms were acquired by heating the sample and a reference aluminium capsule (empty capsule) from 10 to 150 °C at a heating rate of 10 °C/min in a nitrogen atmosphere (flow rate, 100.0 mL/min). Onset, peak, and conclusion temperatures of gelatinization (T_o_, T_p_, and T_c_) along with the gelatinization enthalpy (ΔH, J/g) were quantified using Universal Analysis from Advantage Q Series software 5.1 version (ΤA Instruments, New Castle, DE, USA).

### 2.7. Thermogravimetric Analysis

A SDT Q600 device (TA Instruments, Wakefield, MA, USA) was employed for the thermogravimetric analyses carried out from 30 to 600 °C at 10 °C/min. The analysis was performed for about 5–10 mg of every sample; samples were placed in a non-reactive Pt pan under N_2_ purge flow at 100 mL/min. Percent weight loss was calculated by the percent weight difference between 270 °C and 350 °C.

### 2.8. X-ray Diffraction Analysis (XRD)

The X-ray diffraction tests were performed on the samples in powder form in a Bruker D8 Advance X-ray diffractometer (Bruker, Manning Park Billerica, MA, USA) with monochromatic CuKα radiation at 40 kV and 30 mA. A scanning range (2θ) of 5 to 40° with a step size of 0.05° and a counting time of 15 s per step were applied. The crystallinity degree (CD) was estimated from the area of crystalline peaks divided by the global area, and multiplied by 100 [18,19].

### 2.9. Rheological Analysis

The samples were heated to 83 °C at 5 °C/min, and then the cooled rheological properties of the pastes were assessed after starch suspensions (5%, (*w*/*w*)) stirred at 160 rpm were first cooled at 3 °C/min to room temperature. The flow behavior and dynamic viscoelastic properties were evaluated by means of an AR2000 rheometer (TA Instruments, New Castle, DE, USA) employing a 40 mm diameter parallel plate geometry with a gap of 0.5 mm. The temperature was kept at 20.0 °C using a Peltier system.

Flow behavior was determined in starch pastes through rotational experiments obtaining the viscosity (η) of the samples after achieving the steady state as a function of shear rate ($\dot{\gamma }$) (0.01–1000 s^−1^). Experimental data were fitted to the Carreau–Yasuda model:

$$\eta \left(\dot{\gamma }\right)=\eta_{\infty }+\frac{\left(\eta_{0}-\eta_{\infty }\right)}{{\left[1+{\left(\lambda \cdot \dot{\gamma }\right)}^{a}\right]}^{\frac{1-n}{a}}}$$

where $\dot{\gamma }$ is the shear rate (s^−1^); $\eta_{0}$(Pa·s) is the zero-shear-rate viscosity; $\eta_{\infty }$ (Pa·s) is the high-shear-rate viscosity; λ (s) is the characteristic time constant for the fluid that determines the shear rate at which the transition from the zero-shear-rate plateau to the power-law region takes place; n is the flow index that describes the slope of the power-law region; and a is a dimensionless constant affected by the shape of the zero-shear rate-to-power-law transition.

Strain sweep tests (1 Hz at 25 °C) were first performed to determine the linear viscoelastic region (LVR) of samples. Then, frequency sweep tests were performed from 0.1 to 10 Hz at 25 °C, and the mechanical spectra were obtained. Thus, elastic (G′) and viscous (G″) moduli, as well as the loss tangent (tan δ = G″/G′), were determined.

### 2.10. Film Fabrication

Simple films were fabricated by casting from an aqueous gelatinized solution of the modified starches DS-T and GS-T (FDS-T, FGS-T, respectively) or from an untreated starch (FDS-U) (5% *w*/*w*) as a control system. Films were produced following the procedure informed by Otálora et al. [7]. The suspensions also containing glycerol (2% *w*/*w*), potassium sorbate (0.2% *w/w*), and distilled water up to 100 g were mixed (400 rpm) under heating (rate: 5 °C/min) up to 83 °C. Each film-forming solution (24 g) was cast into silicone molds (diameter = 7 cm) (Silikomart, Venice, Italy) and dried in a controlled temperature chamber at 45 °C (FAC, Buenos Aires, Argentina) for 15 h. Once the films were constituted, they were stabilized over a saturated NaBr solution (water activity, a_w_ ≅ 0.575) for 5 days at 25.0 ± 0.5 °C, before characterization.

#### 2.10.1. Characterization of the Films

##### Water Uptake Capacity and Soluble Matter Loss

Water uptake capacity (WUC) tests were performed following a modified protocol based on the ASTM D570 [20]. Soluble material loss (SML) was estimated according to Otálora et al. [19].

##### Contact Angle and Water Vapor Permeability (WVP)

The contact angle was evaluated with an interfacial tensiometer (Sinterface, Berlin, Germany) using the sessile drop method according to Otálora et al. [7]. The WVP was determined twice gravimetrically at 25 °C, using the procedure established in ASTM E96-00 [21], adapted by Gennadios et al. [22] for hydrophilic films.

##### Mechanical Properties

Tensile properties were determined at room temperature in a universal Instron testing machine 3345 (Instron Corp, Norwood, MA, USA), equipped with a 100 N load cell and a strain rate of 0.8 mm/s, The method described by the D882-02 standard [23] was followed with some modifications. Stress–strain curves were first obtained, and then characteristic tensile parameters were calculated: maximum stress (σ_max_) and strain at break (ε_max_). Ten replicates for every film formulation were analyzed.

#### 2.10.2. Scanning Electron Microscopy (SEM)

The microstructure of the films was observed employing a Scanning Electron Microscope ZE122 SEM Supra 40 (Carl Zeiss, Jena, Germany). The cross-sections and surface of the films were gold-coated before any observation. Images analysis was conducted with SmartSEM^®^ V05.06 software (Carl Zeiss, Jena, Germany).

### 2.11. Experimental and Statistical Analysis

The Prism 5 software (GraphPad, Boston, MA, USA) was used for non-linear fits and statistical evaluations. The statistical significance was evaluated using ANOVA with a level α of 0.05. The Tukey test was used as an “a posteriori test” [24]. All measurements of starch samples and edible films were performed at least three times, with results reported as averages and standard deviations.

## 3. Results and Discussion

The CT at atmospheric pressure is an unconventional procedure for the chemical modification of cassava starch. During plasma treatment, different changes can occur: starch cross-linking, starch de-polymerization, and modification of the surface (damages) of starch granules, all of these due to the action of the energy evolved from the process as well as due to the species present in the plasma generated [5,6,15,25]. According to the prevalence of these reactions, different effects can be generated affecting the characteristics and properties of the starches involved. The form of presentation of starch samples to CT as well as the experimental arrangement used for the CT can also exert an important influence on the trends observed.

### 3.1. Starch Characterization

#### 3.1.1. FTIR

**Figure 1:**
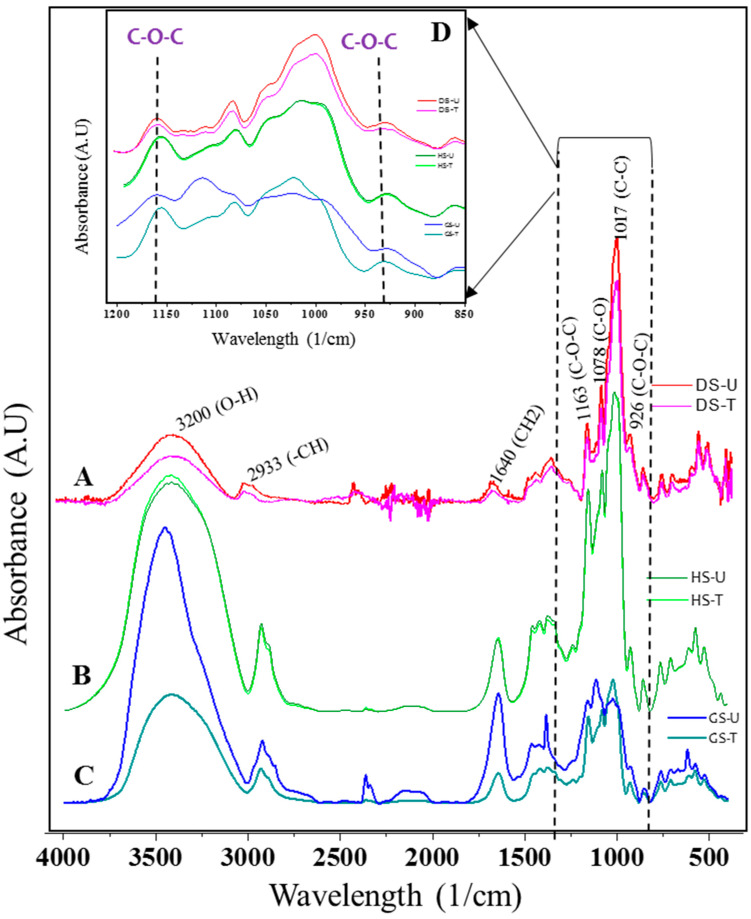
FTIR spectra of untreated and treated samples of starch. (**A**) DS-U versus DS-T, (**B**) HS-U versus HS-T, (**C**) GS-U versus GS-T, and (**D**) changes in C-O-C band intensities between DS-T and GS-T with respect to DS-U and GS-U.

Figure 1 shows the FTIR spectra of untreated and plasma-treated starch samples. The starch samples had three main IR peaks at 3100–3600 cm^−1^ (O-H stretching), 3000–2800 cm^−1^ (C-H stretching), and 1640 cm^−1^ (O-H bending of bound water) [11,19]. Asymmetric C-O-C, C-O, and C-C skeleton stretching vibrations lead to the occurrence of absorption peaks at 1163 cm^−1^, 1089 cm^−1^, and 1017 cm^−1^, respectively. On the other hand, the peak at 926 cm^−1^ was due to the skeletal mode vibrations of the C-O-C α-1,4-glycosidic bonds. Nemţanu and Braşoveanu [25] and Gao et al. [11] reported similar peaks. No new absorption peaks were distinguished for starches after plasma treatment.

A decrease in the intensity of OH groups is observed in the FTIR spectra, as seen from the peak at wavenumber 3200 cm^−1^ and 1640 cm^−1^ after plasma treatment for the DS-T and GS-T samples (Figure 1A,C). The decrease in the bands indicates the loss of OH groups in the modified starch, which would indicate that a plasma-induced cross-linking mechanism occurred. This mechanism forced an O-H bond and a C-OH bond to break before cross-linking, generating a new C-O-C glycosidic bond between two starch chains and extracting a water molecule. This would explain the decrease in OH groups. Khorram et al. [9] reported a decrease in the OH group in modified starch using argon and oxygen glow discharge plasma, revealing that cross-linking occurred.

In DS-T and GS-T samples, changes in the intensities of the bands at 1163 cm^−1^ and 926 cm^−1^ were observed (Figure 1D), probably indicating the modifications of C-O-C bonds, which could mean that starch cross-linking by plasma treatment is the prevailing reaction for the occurrence of these bonds. The cross-linking of the starch molecules because of the increase in the glycosidic bond was confirmed by an increase in the absorption peak at 926 cm^−1^ in cassava starches. Free radicals and energetic electrons formed during plasma generation may induce that cross-linking or intercrossing among the starch molecules [26]. Zou et al. [27] previously reported the cross-linking of starch in argon glow discharge plasma with a mechanism like that reported before.

The FTIR spectra of the HS-T samples were like the control sample (HS-U) (Figure 1B). According to Guo et al. [28], water can be activated by means of plasma application and can be used to enhance agriculture production. Therefore, the energy generated by corona electrode might have been at least partially used by the water present in this system to be activated, diminishing the availability of energy and active species to perform changes in the studied starch.

#### 3.1.2. pH, Water Retention Capacity (WRC), and Solubility in Water (S)

After the plasma treatment, a lowering in the pH of the starch solution (Table 1) was detected, indicating the formation of chemical groups with acidic character, such as carboxyl, carbonyl, or peroxide groups [4], due to the oxidation of starch. However, in the FTIR spectrum (Figure 1), no observable change was detected for the peak at around 1700 cm^−1^, which corresponds to the carboxylic acid functional group. Thirumdas et al. [13] have previously reported that the decrease in pH could not be confirmed from the changes in FTIR spectra of rice starch.

It was observed that there was a significant decrease in WRC and S in the DS-T and GS-T modified starch when compared with untreated starch (DS-U, GS-U) (Table 1). A decrease in the water solubility of corn starch was observed using corona discharge plasma by Nemtanu and Minea [29], and this decrease could be attributed to cross-linking between starch molecules due to the effect of the active species of the plasma treatment that reduced the number of hydroxyl groups, producing starch with a lower capacity to interact with water [4]. The reduction in –OH groups could be evidenced by the significantly lower-intensity bands of O-H stretching for DS-T and GS-T samples in FTIR spectra (Figure 1A,C).

In contrast, HS-T showed an increase in S and WRC after treatment (Table 1). This could be attributed to the de-polymerization of starch, which could lead to the formation of smaller fragments that have higher water absorption power, resulting in higher solubility [10]. In addition, the CT energy could cause surface damage to the starch granules, which might have enhanced water penetration, resulting in higher WRC and S. Thirumdas et al. [14] demonstrated that cold air plasma increased the water absorption rate and solubility of rice starch.

The different trends observed in this study for cold plasma application could be attributed to the different presentation of samples used as well as to different treatment conditions, such as the type of electrode used for the different samples. It is important to remark that plasma in contact with water also promotes the formation of water with chemically reactive oxygen and nitrogen species that can modify the starch properties [29,30].

#### 3.1.3. Pasting Properties

**Figure 2:**
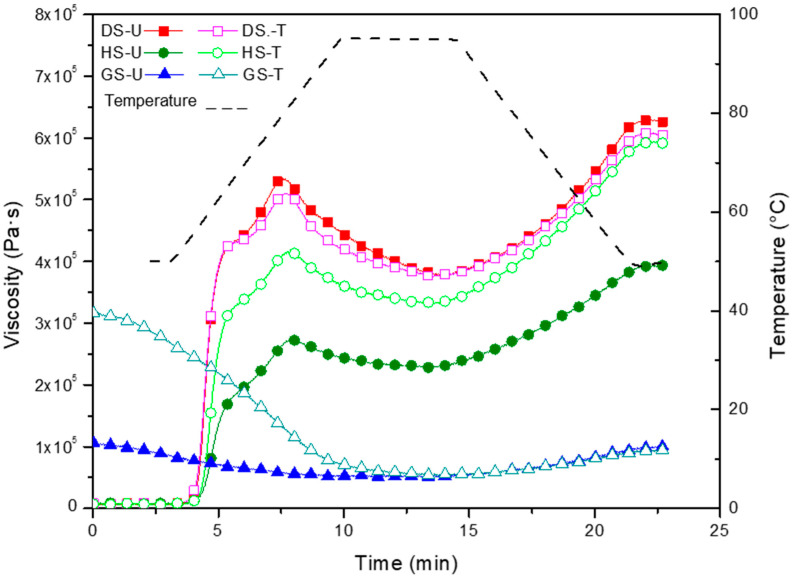
Pasting curves of untreated and plasma-modified starches.

Pasting of starch involves different phenomena, such as granular swelling, transformation from ordered to disordered state, exudation of certain molecular components, and finally, the disruption of the granules [31,32]. The processing quality of starch depends greatly on its pasting properties. For example, understanding pasting behavior can be used to optimize the concentration of the ingredients, as well as the processing conditions (i.e., temperature, pressure, shear limits) when manufacturing the desired product [31]. Pasting properties are often determined from pasting curves obtained through a rapid visco-analyzer (RVA), which is a temperature-controlled viscometer that monitors the resistance of a starch granule sample to a specific shear. The pasting curves of the untreated (DS-U, HS-U, GS-U) and treated (DS-T, HS-T, GS-T) cassava starch samples are shown in Figure 2 and their pasting parameters are summarized in Table 2.

The pasting temperature (PT) indicates the temperature at which the viscosity abruptly increases during the continuous heating process. The PT did not change after CT for DS-T and HS-T (Table 2).

Regarding dry starch, plasma treatment significantly reduced the peak viscosity (PV, maximum viscosity during pasting) of dry starch from 5.34 × 10^5^ Pa·s (DS-U) to 5.10 × 10^5^ Pa·s (DS-T), as the energy involved in the treatment probably produced the modification of the starch macromolecules [5]. This should also be associated with the decrease observed in swelling capacity (Table 1). A significant decrease in breakdown viscosity (BV) was also detected in the treated starch (DS-T). BV is used to evaluate the shear thinning response of starches [10]. As displayed in Table 2, plasma-treated DS starches showed a good resistance to shear thinning when heated because cross-linking would have prevailed after plasma treatment. Chaiwat et al. [33] reported that the formation of covalent cross-links between starch chains strengthened the swollen granules, which minimized the viscosity loss under high temperature and shear conditions. Table 2 shows that SV and FV in DS-T samples decreased significantly (*p* < 0.05) compared to untreated starch (DS-U). Setback values (SVs) are defined as the degree of re-association between starch molecules involving amylose [11]. Zhang et al. [34] observed a global reduction in viscosity after potato starches were processed with nitrogen and helium glow plasma.

In humid starch, there was an increase in viscosities after treatment (Table 2). An increase in the viscosity of rice starch after cold plasma treatment was reported by Thirumdas et al. [14], as previously mentioned. Maximum viscosity represented the equilibrium point between swelling and breaking of starch granules. The increase in PV correlates with an enhancement in solubility and WRC after plasma treatment (HS-T) (Table 1). The higher FV in HS-T during the cooling process could be because amylose began to retrograde and the bonds between the chain molecules were reformed. In addition, the high-energy plasma species result in some degree of molecular alteration of the starch that altered the final viscosity [14,35].

The treatment of gelatinized starch with plasma (GS-T) increased significantly both BV and SV (Table 2). The incorporation of water molecules is facilitated during starch modification, resulting in increased viscosities (BV and SV). Therefore, plasma treatment causes de-polymerization of starch molecules due to the breaking of hydrogen bonds [14,33].

#### 3.1.4. Gelatinization Properties

DSC analysis was performed to determine the gelatinization properties of DS-U, DS-T, HS-U, and HS-T samples. Since GS-U and GS-T samples were already gelatinized, the DSC technique was not applied. The onset, peak, and conclusion temperature (T_o_, T_p_, and T_c_) of DS-T and HS-T increased with plasma treatment (Table 2). Bie et al. [35] reported an increase in the gelatinization temperature of cassava starch treated with helium plasma. A decrease in gelatinization enthalpy (ΔH) was detected after treatment for DS, being 81 and 34 J/g for DS-U and DS-T, respectively. In the case of wet starch, the decrease in ΔH was more drastic, from 80.3 to 0.63 J/g for HS-U and HS-T, respectively (Table 2). Some degree of crystallinity is probably lost during treatment. The decrease in enthalpy shows that plasma-treated starches consume less energy for gelatinization. Wang et al. [36] reported that the gelatinization enthalpy (ΔH) shows the loss of the double helix structure of starch molecules. Carmona-Garcia et al. [37] produced the cross-linking of banana starch with sodium trimetaphosphate (STMP)/sodium tripolyphosphate (STPP) and demonstrated an increase in gelatinization temperatures with cross-linking accompanied by a slight decrease in ΔH.

#### 3.1.5. Thermogravimetric Analysis

**Figure 3:**
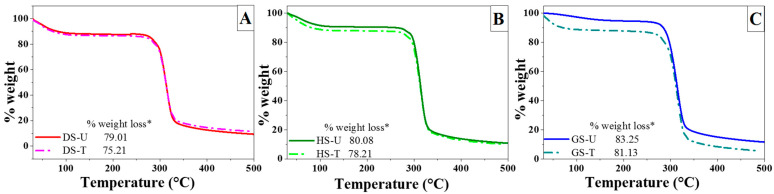
TGA thermograms and %weight loss of the untreated starches (DS-U, HS-U, and GS-U) and plasma-treated starches (DS-T, HS-T, and GS-T). * %weight loss = %weight at 270 °C − %weight at 350 °C. (**A**) DS-U and DS-T. (**B**) HS-U and HS-T. (**C**) GS-U and GS-T.

Figure 3 shows the weight loss curves when untreated starches and plasma-treated starches were heated at 10 °C/min for a period of time. The TGA profiles of the starch samples show a region of weight loss in the range of 270 to 350 °C, which refers to the thermal decomposition of starch [15]. These results showed that the weight losses of plasma-modified starches (DS-T, HS-T, and GS-T) were lower than those of unmodified starches (DS -U, HS -U, and GS -U) (Figure 2), which could be due to the prevalence in the results observed of cross-linking reactions that took place during plasma treatment. This implies a better thermal stability, a relatively good thermal stability, and a relatively stronger structure after plasma treatment, which could be associated with a cross-linking reaction of the modified starch. Singh and Nath [38] reported that cross-linked sago starch exhibited a higher thermal stability when compared to native sago starch.

#### 3.1.6. RX Diffraction

**Figure 4:**
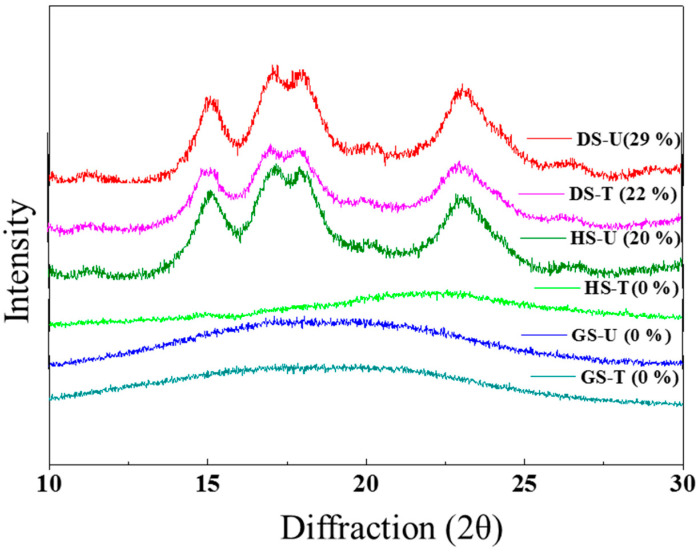
X-ray diffraction patterns and degree of crystallinity (DC) (in parentheses) of starch samples.

The XRD patterns of cassava starches before and after plasma treatment are shown in Figure 4. Characteristic X-ray diffraction peaks, which correspond to the A-type crystal pattern of cassava starch [18,39], at 2θ of 15°, 17°, 19°, and 23° appear in samples DS-U, DS-T, and HS-U. The crystallinity slightly decreases with plasma treatment in DS-T. It was observed that the % degree of crystallinity (DC) decreased from 29% (untreated starch) to 22% (DS-T) after treatment in agreement with DSC results. The decrease in crystallinity is due to the loss of ordered structures of starch molecules caused by reactive species in plasma [34]. When studying the influence of oxygen and helium plasma on the crystallinity of cassava starch, Bie et al. [35] observed an 8% decrease in the DC after helium gas plasma treatment, while a 12% decrease was observed after nitrogen gas plasma. In the present study, a 7% decrease in CD after atmospheric air plasma treatment was observed in the dried starch samples. It can be concluded that both the treatment time and the type of feed gas employed for plasma generation affect the rate of decrease in crystallinity.

**Figure A1:**
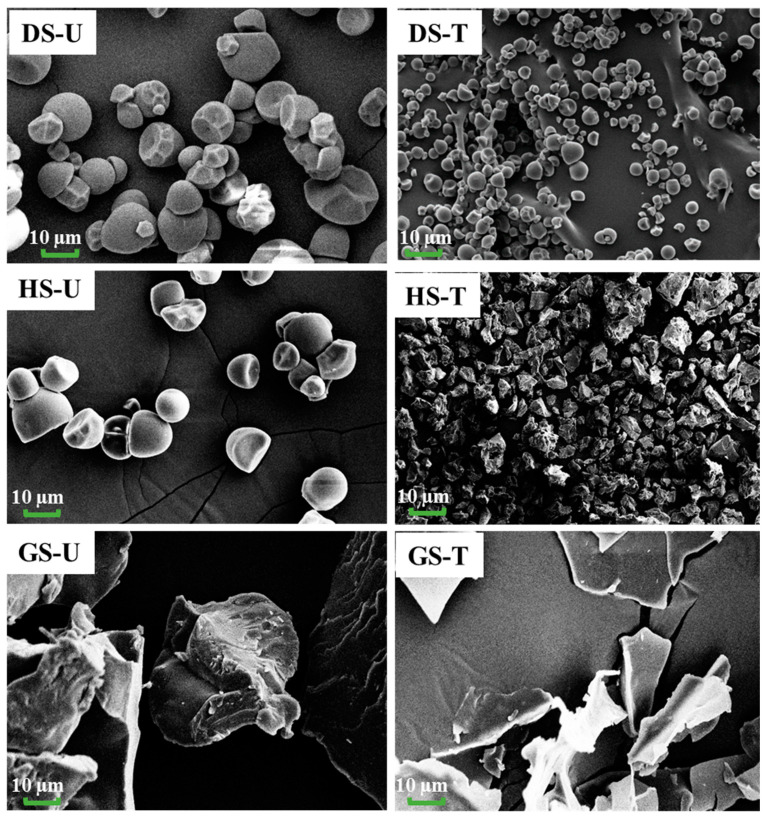
SEM images of the untreated and treated starch samples.

In systems where the starch granule was treated in suspension with water (HS-T), the absence of XRD patterns was observed after treatment. This change could be due to an increase in temperature during CT that had determined important progress in the gelatinization of the starch granule (Figure A1 shows the SEM images of the starch granules employed), as could also be inferred from a very low ΔH value (Table 2) for this sample. Previous work reported a reduction in rice starch crystallinity after plasma treatment [6,40]. The decrease in the degree of crystallinity of modified starch DS-T and HS-T coincides with the decrease in the gelatinization enthalpy (ΔH) (Table 2), that is, a lower energy requirement for starch gelatinization. Thus, plasma treatment affects the amorphous and crystalline regions of starch molecules.

#### 3.1.7. Rheological Properties of Starch Paste

**Figure 5:**
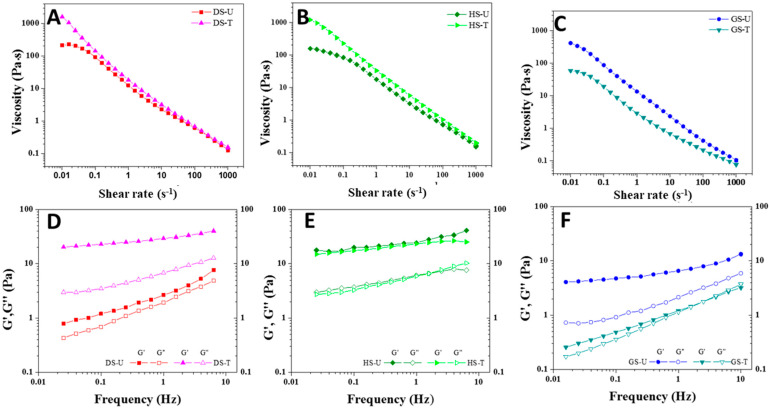
Flow curves (**A**–**C**) and mechanical spectra (**D**–**F**) for untreated and plasma-modified starches.

The study of the rheological parameters is generally of great importance for the starch industries. In this section, the rheological properties of the pastes obtained after the sample preparation described in Section 2.9 are analyzed. Thus, the viscosity as a function of the shear rate for every sample is displayed in Figure 5A–C. As expected for shear-thinning fluids, viscosity decreased as shear rate increased, which can be explained on the basis of a structural rearrangement. So, intra- and intermolecular bonding in the starch network is disrupted under shear stress, and then aggregates can align in the direction of the force through rearrangement or deformation. This would finally result in a reduction in the internal resistance and a viscosity reduction [14]. The DS-T and HS-T curves (Figure 5A,B) showed the highest viscosity throughout the shear rate range. In contrast, the GS-T samples (Figure 5C) displayed lower viscosity values than GS-U because of CT. It has been reported that the viscosity of starch was either increased or decreased by plasma treatment, depending on the types of starch and plasma as well as the treatment conditions [29,35]. In this study, the Carreau-Yasuda model was used to fit the flow curves of starch pastes with a coefficient of determination (R^2^) between 0.9975 and 0.9983. The rheological parameters obtained for plasma-treated starches after fitting to the Carreau-Yasuda model are presented in Table 3. All starch samples showed pseudoplastic behavior, with values of flow index n less than 1. The Newtonian zero-shear-rate viscosity (η_ο_) of (DS-U) showed a value of 220.4 Pa·s and that of DS-T showed a significantly higher value of 1992.7 Pa·s, demonstrating that plasma treatment of dry starch granules probably produced a cross-linking phenomenon that not only increased η_ο_, but also increased the relaxation time (λτ_c_) (Table 3). This same trend was observed for the samples that were humid-treated (HS-T). On the other hand, samples that were first gelatinized and then treated with plasma showed the opposite behavior, as a decrease in the flow curve parameters was observed after treatment.

Small Amplitude Oscillatory Shear (SAOS) tests provide information of the elastic modulus (G′) and viscous modulus (G″) as a function of frequency (i.e., mechanical spectra), which can be related to the sample structure. All analyzed paste samples could be classified as weak gels because G′ > G″ within the frequency range studied and the moduli showed a slight slope (Figure 5D–F). The G′ and G″ of the treated starch DS-T showed higher values of moduli than the untreated dry starch (DS-U) (Figure 5D), whereas those of the HS-U paste were almost the same as those of the treated (HS-U) paste (Figure 5E). In addition, the DS-T paste showed a lower magnitude of loss tangent (tan δ) than the control (DS-U) paste (Table 3). This would indicate a stronger gel structure for the treated sample (DS-T), probably due to cross-linking reactions. This is supported by the greater dependency of G′ and G″ on frequency found for the DS-U sample, even finding a tendency for a crossover at frequencies higher than 1 Hz. This is not observed for the DS-T sample, for which much higher values for both viscoelastic moduli are found, with a much more prevailing elastic character. The gelatinization of the starch followed by the CT is responsible of this strengthening, which is coherent with the flow behavior previously studied. Chaiwat et al. [33] found that G′ of starch cross-linked by low-pressure argon plasma treatment was higher than that of native starch. In general, an increase in G′ accompanied by a decrease in tan δ could be related to cross-linking formation of the polymer [15].

### 3.2. Characterization of the Films

In general, starch is used in the manufacture of edible films due to its ability to form flexible films and its acceptable barrier to gases such as oxygen and carbon dioxide. The technological application of these films has been restricted due to some inherent disadvantages, such as inadequate mechanical and water vapor barrier properties, high water sensitivity, and poor printability and adhesiveness. Therefore, it is necessary to use different treatments to improve its characteristics, such as CT. As previously stated, DS-T and GS-T modified starches showed lower WRC and S values in comparison with ones for untreated samples, probably due to the predominance of cross-linking reactions promoted by TC. The opposite was the case with the modified HS-T starch, where there was a higher WRC and S after CT. Therefore, DS-T and GS-T modified starches were selected to produce edible films based on their physicochemical characteristics.

#### 3.2.1. Water Vapor Permeability, Water Uptake Capacity, and Contact Angle of Films

**Figure 6:**
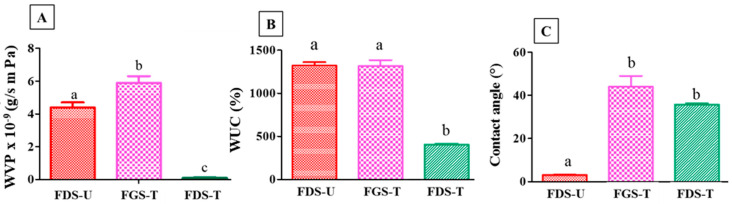
(**A**) Water vapor permeability (WVP), (**B**) water uptake capacity (WUC), and (**C**) contact angle of films (FDS-U) and treated films (FGS-T and FDS-T). The average and standard deviation are reported. Same letter means non-significant differences (*p* < 0.05) between samples.

One of the main restrictions of the application of cassava starch films is their mostly hydrophilic nature, which leads to high water transfer rates due to the clustering of water molecules and their diffusion through microcavities. The WVP for the control cassava starch film (FDS-U) was found to be 4.4 ± 0.3 × 10^9^ g s^−1^ m^−1^·Pa^−1^. When films were manufactured from GS-T, a significant increase (*p* > 0.05) in WVP was observed, whereas when DS-T was used, there was a very marked decrease in WVP (Figure 6A). These results coincide with the decrease in WUC in the FDS-T (Figure 6B). This decrease may be due to the reduction of hydrophilic groups (-OH) observed in FTIR analyses (Figure 1A) and to the polymeric morphological characteristics in addition to starch cross-linking. In previous studies, a decrease in WVP of 58% was observed when the surface of cassava starch-based films was modified with CT [7]. Plasma treatment decreased the WVP of starch/PCL and starch/PLA composite films by approximately 94% according to Heidemann et al. [41].

The contact angle of a liquid is related to its wettability and hydrophilicity, as it measures the tendency of a drop of that fluid to spread and adhere to the surface of materials. The FDS-U showed a contact angle of 3° (Figure 6C). When FGS-T and FDS-T were produced, the contact angle increased significantly to a value of 44° (FGS-T) or to a value of 35.7° (FDS-T), which means that the hydrophilicity of the surface of the film was lower (Figure 6C), probably due to the changes suffered by the starch after CT [7]. Lyytikäinen et al. [42] reported that oxidation compounds can be formed during treatment (i.e., hydroxyl groups of starch can be oxidized to carbonyls), increasing the hydrophobic character of the polysaccharide and of the films constituted with it. Pankaj et al. [43] used plasma treatments on cassava starch films and observed an increase in hydrophobicity. Bastos et al. [44] showed that the contact angle increased after CT and more hydrophobic groups were obtained in the surface layer of the starch films.

#### 3.2.2. Physical, Mechanical, and Morphological Properties

Table 4 shows the physical and mechanical properties of the films. A decrease in moisture content is observed in FDS-T compared to the control (FDS-U). This might be ascribed to the cross-linking of starch, thus limiting the water sorption capacity, and coincides with the decrease in WUC in the FDS-T. Solubility in water is an important property of edible films. Potential applications may require insolubility to improve product integrity and water resistance. In Table 4, SML is lower in FDS-T and FGS-T with respect to FDS-U, which indicates greater water resistance. This result coincides with the decrease in WUC (Figure 6C). The energy of the treatment probably promoted the cross-linking of the starch macromolecules [5], resulting in strengthened structure with poor solubility.

The mechanical characteristics of edible films are also key factors in protecting the quality and integrity of food products during different stages (i.e., handling, transportation, storage). Results obtained indicated that the FGS-T films had weak mechanical properties since the stress values decreased from 1.62 to 1.3 MPa. In the FDS-T films, an improvement in mechanical resistance was observed since the stress values increased from 1.62 to 2.0 MPa (Table 4). Guo et al. [45] treated potato starch with dielectric barrier discharge plasma treatment and observed that this treatment gave rise to films with better tensile stress and Young’s modulus.

**Figure 7:**
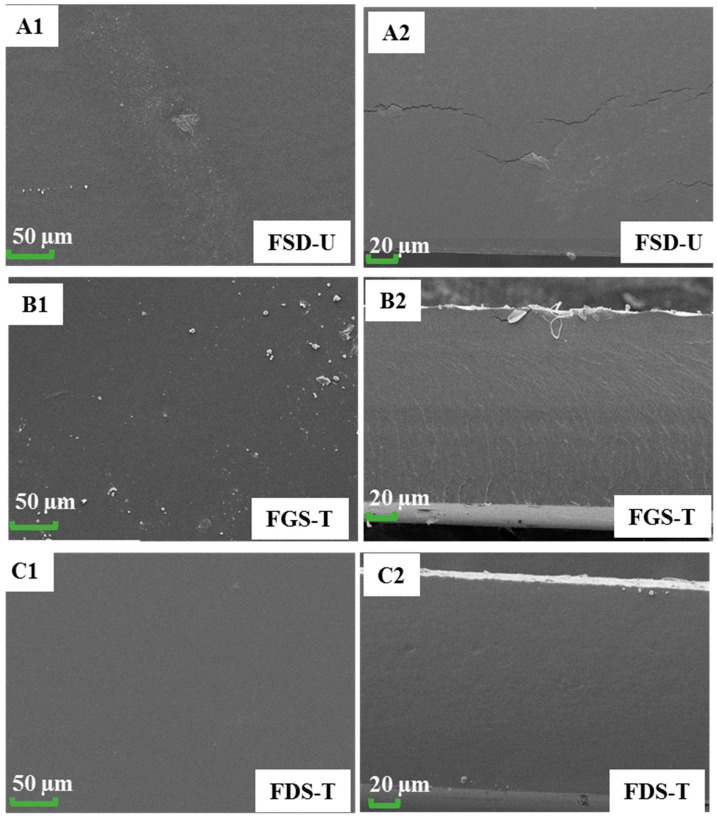
SEM images of the surface (**A1**,**B1**,**C1**) and cross-section (**A2**,**B2**,**C2**) of FDS-U, FGS-T, and FDS-T.

Cracks can be observed in the surface and cross-section micrographs of control films, FDS-U (Figure 7(A1,A2)), which explained the higher WVP and WUC and the lower contact angle. Meanwhile, FGS-T (Figure 7(B1,B2)) and FDS-T (Figure 7(C1,C2)) showed a compact structure, without cracks and phase separation, which can explain the improvement in stress in the FDS-T systems. The observed morphological changes could be a consequence of starch cross-linking [5]. Other researchers reported similar trends in relation to the results of the use of plasma-treated starch on the production of films [10]. An improvement in structural properties of films based on plasma-treated starch has been attributed to the effect of the bombardment of plasma species such as electrons, ions, and radicals on the polysaccharide, favoring its cross-linking [5,43,45]. Similarly, this may explain the increase in contact angle, which means an increase in hydrophobicity (Figure 6C).

## 4. Conclusions

Results highlight corona treatment (CT) at atmospheric pressure as a useful alternative to chemical modifications commonly employed for cassava starch. CT produces changes in the functional, thermal, and rheological properties of starch. The study of the behavior of films produced through casting from plasma-modified cassava starch showed notable improvements in their mechanical properties when compared to those of native starch films, as they were more resistant and had lower water absorption capacity and greater hydrophobicity. Changes also occurred in the structure of the films when observed by SEM, contributing to the generation of a more compact and homogeneous structure when films were based on CT-modified starch. Thus, CT is a sustainable technology that enables the industrial processing of edible films based on modified cassava starch with adequate properties. These new materials could be a viable and environmentally friendly alternative to be used as food packaging material.

Future investigations must consider (i) surface analysis of the films developed by XPS to evaluate the surface modifications produced by atmospheric CT, (ii) evaluation of the ability of the developed films to carry pigments and antimicrobial and antioxidant compounds. All of this will help to determine the specific applications of CT treatment. The application of CT to other starches is also of interest to analyze whether the conclusions obtained for these treatments can be generalized.

## Figures and Tables

**Table 1:** pH, water retention capacity (WRC), and solubility (S) of treated and untreated starches.

Sample	pH	WRC (g/g)	S (%)
DS-U	6.49 ± 0.04 ^a^	18.8 ± 0.8 ^a^	26 ± 1 ^a^
DS-T	4.87 ± 0.06 ^b^	9.10 ± 0.4 ^b^	6.2 ± 0.4 ^b^
HS-U	5.67 ± 0.04 ^a^	18.2 ± 0.5 ^a^	14 ± 1 ^a^
HS-T	3.45 ± 0.07 ^b^	22.0 ± 1.0 ^b^	20 ± 2 ^b^
GS-U	5.68 ± 0.03 ^a^	11.5 ± 0.6 ^a^	42 ± 2 ^a^
GS-T	3.89 ± 0.02 ^b^	9.50 ± 0.6 ^b^	37 ± 1 ^b^

Data are expressed as mean ± SD. Means between untreated and treated starches (DS-U vs. DS-T, HS-U vs. HS-T, and GS-U vs. GS-T) with different letters in the same column are significantly different (*p* < 0.05).

**Table 2:** Pasting properties and gelatinization properties of the untreated and corona electrical discharge-treated cassava starches.

Samples	Pasting Properties					Gelatinization Properties			
	PT (°C)	PV × 10^3^ (Pa·s)	BV × 10^3^ (Pa·s)	FV × 10^3^ (Pa·s)	SV × 10^3^ (Pa·s)	T_o_ (°C)	T_p_ (°C)	T_c_ (°C)	∆H (J/g)
DS-U	67.9 ± 0.5 ^a^	534 ± 1 ^a^	156.4 ± 0.8 ^a^	625 ± 1 ^a^	91.1 ± 0.8 ^a^	61.3 ± 0.5 ^a^	69.4 ± 0.7 ^a^	84.7 ± 0.9 ^a^	81 ± 1 ^a^
DS-T	66.5 ± 0.2 ^a^	510 ± 2 ^b^	135 ± 2 ^b^	604 ± 2 ^b^	93.7 ± 0.1 ^b^	82.0 ± 1.0 ^b^	85.0 ± 2.0 ^b^	96.0 ± 1.0 ^b^	34 ± 2 ^b^
HS-U	68.5 ± 0.4 ^a^	273.9 ± 0.3 ^a^	45.8 ± 0.4 ^a^	391 ± 1 ^a^	117.8 ± 0.7 ^a^	58.9 ± 0.4 ^a^	63.9 ± 0.7 ^a^	73.3 ± 0.5 ^a^	80.3 ± 0.6 ^a^
HS-T	67.9 ± 0.8 ^a^	415.7 ± 0.5 ^b^	84.4 ± 0.8 ^b^	592 ± 1 ^b^	176.7 ± 0.5 ^b^	62.6 ± 0.7 ^b^	68.8 ± 0.8 ^b^	76.8 ± 0.4 ^b^	0.63 ± 0.03 ^b^
GS-U	ND	ND	51.9 ± 0.5 ^a^	99.1 ± 0.7 ^a^	3.61 ± 0.04 ^a^	ND	ND	ND	ND
GS-T	ND	ND	257.4 ± 0.8 ^b^	94.1 ± 0.6 ^b^	216.7 ± 0.7 ^b^	ND	ND	ND	ND

ND, not detectable; PT, pasting temperature PV, peak viscosity; BV, breakdown viscosity; FV, final viscosity; SV, setback viscosity; T_o_, onset temperature; T_p_ peak temperature; T_c_, conclusion temperature; ΔH, enthalpy of gelatinization. Data are expressed as mean ± SD. Means of untreated and treated starches (DS-U vs. DS-T, HS-U vs. HS-T, and GS-U vs. GS-T) with different letters in the same column are significantly different (*p* < 0.05).

**Table 3:** Rheological properties of the untreated and corona electrical discharge-treated cassava starches.

Samples	Steady Flow Test						Dynamic Viscoelastic Test		
	η_0_(Pa·s)	η_∞_ (Pa·s)	λ (s)	n	a	R^2^	G′_1 Hz_(Pa)	G″_1 Hz_ (Pa)	tan δ_1 Hz_
DS-U	220.4 ± 0.7 ^a^	0.072 ± 0.001 ^a^	39.4 ± 0.8 ^a^	0.250 ± 0.07 ^a^	8.08 ± 0.3 ^a^	0.9975	3.2 ± 0.8 ^a^	2.1 ± 0.3 ^a^	34 ± 1 ^a^
DS-T	1992.7 ± 0.2 ^b^	0.123 ± 0.04 ^b^	220.0 ± 0.2 ^b^	0.146 ± 0.002 ^b^	24.3 ± 0.1 ^b^	0.9962	29 ± 0.3 ^b^	6.9 ± 0.3 ^b^	13.2 ± 0.2 ^b^
HS-U	157.3 ± 0.3 ^a^	0.048 ± 0.003 ^a^	19.9 ± 0.5 ^a^	0.27 ± 0.01 ^a^	1.52 ± 0.05 ^a^	0.9993	6.1 ± 0.4 ^a^	2.7 ± 0.1 ^a^	24.1 ± 0.6 ^a^
HS-T	1212.3 ± 0.5 ^b^	0.058 ± 0.005 ^a^	84.4 ± 0.3 ^b^	0.24 ± 0.01 ^a^	14.5 ± 0.5 ^b^	0.9997	21.7 ± 0.7 ^b^	5.6 ± 0.5 ^b^	14.4 ± 0.2 ^b^
GS-U	302.4 ± 0.2 ^a^	0.047 ± 0.006 ^a^	63.4 ± 0.5 ^a^	0.22 ± 0.02 ^a^	4.44 ± 0.4 ^a^	0.9998	6.2 ± 0.2 ^a^	2.2 ± 0.1 ^b^	20.3 ± 0.7 ^a^
GS-T	22.52 ± 0.8 ^b^	0.048 ± 0.001 ^a^	33.9 ± 0.3 ^b^	0.39 ± 0.06 ^b^	68.0 ± 0.1 ^b^	0.9983	1.5 ± 0.4 ^b^	1.3 ± 0.2 ^b^	41.3 ± 0.3 ^b^

Data are expressed as mean ± SD. Means between untreated and treated starches (DS-U vs. DS-T, HS-U vs. HS-T, and GS-U vs. GS-T) with different letters in the same column are significantly different (*p* < 0.05).

**Table 4:** Moisture, solubility in water, and mechanical parameters of control films (FDS-U) and treated films (FGS-T and FDS-T).

Physical Parameters	FDS-U	FGS-T	FDS-T
Moisture (% db)	24.7 ± 0.2 ^a^	23 ± 1 ^a^	20.2 ± 0.1 ^b^
SML (%)	40.1 ± 0.7 ^a^	35 ± 2 ^b^	32 ± 1 ^b^
**Mechanical properties**			
ε_max_ (mm/mm)	1.25 ± 0.07 ^a^	1.1 ± 0.1 ^a^	1.4 ± 0.1 ^a^
σ_max_ (MPa)	1.62 ± 0.03 ^a^	1.3 ± 0.1 ^b^	2.0 ± 0.2 ^c^

The mean and standard deviation are reported. Same letter in the data reported in a row means non-significant differences (*p* < 0.05).

## Data Availability

The original contributions presented in the study are included in the article, further inquiries can be directed to the corresponding author.
